# Evolutionary Dynamics of Oncosuppression Under Selection Pressure

**DOI:** 10.3390/life15101556

**Published:** 2025-10-03

**Authors:** Mikhail Potievskiy, Peter A. Shatalov, Ilya Klabukov, Dmitrii Atiakshin, Anna Yakimova, Denis Baranovskii, Peter V. Shegai, Andrey D. Kaprin

**Affiliations:** 1 National Medical Research Radiological Center of the Ministry of Health of the Russian Federation, 4 Koroleva Street, 249036 Obninsk, Russia; 2 Obninsk Institute for Nuclear Power Engineering, National Research Nuclear University MEPhI, Studgorodok, 1, 249036 Obninsk, Russia; 3 Department of Urology and Operative Nephrology, Peoples’ Friendship University of Russia (RUDN University), 6 Miklukho-Maklaya Street, 117198 Moscow, Russia; 4 University Hospital Basel, Basel University, 4001 Basel, Switzerland

**Keywords:** cancer, evolutionary medicine, evolutionary oncology, oncosuppressors, mammalian adaptations, vertebrate aromorphoses

## Abstract

**Background and Objectives:** Changes in the environment and physiology may be associated with an increased or decreased risk of cancer. Our study aims to evaluate the strength and the direction of the selection acting on oncosuppressor genes in association with phenotypic changes. **Methods:** We calculated the relative evolutionary rate (RER) using the converge method and linear regression on branches of phylogenetic trees. The association between changes in the evolutionary rate of oncosuppressors (DNA repair and cell cycle control genes) and trait selection was studied. The evolutionary rates of single oncosuppressor genes and pathways were evaluated. We studied two types of traits: those that are characteristic of vertebrates, such as homeothermy (the ability to maintain a constant body temperature), flight, and amnions; and those that are characteristic of mammals, such as high body mass and lifespan, an underground lifestyle, and hibernation. The analysis included 19,445 genes; 100 vertebrates and 46 mammalian species. We studied ancestral branches individually and all the clades having a trait. **Results:** Oncosuppressor genes accelerated in association with the ability to fly; *p*-value = 0.03 (positive or relaxed negative selection) and decelerated in homeothermic species; *p*-value = 0.04 (stabilizing selection). DNA repair genes were significantly accelerated in ancestral branches and in all clades of amniotic, homeothermic, and high-body-mass mammals (*p*-value < 0.05, FDR correction). Cell cycle control genes were under stabilizing selection in homeothermic animals, high-body-mass, long-lived, and underground mammals (*p*-value < 0.05, FDR correction). Data on the evolution of oncosuppressors are crucial for understanding the origin of cancer and will be important for future studies of tumor pathogenesis, pathomorphosis, and microevolution. **Conclusions:** The selection of traits associated with changes in cancer risk leads to positive/relaxed negative and stabilizing selection of oncosuppressor genes.

## 1. Introduction

According to the WHO, cancer is the most common cause of death in developed countries [1]. Effective treatment of any disease requires understanding its origin and development; however, cancer is heterogeneous and associated with different mutation accumulation and gene expression changes. Tumors evolve, and divide into various clones, leading to disease progression and therapy resistance [2,3].

Over 500 million years ago, the transition from unicellular to multicellular life demanded cooperative mechanisms for cellular proliferation, differentiation, and death to ensure organismal survival. However, the breakdown of these cooperative systems gives rise to rogue cells that prioritize their own replication, leading to cancer [4,5,6].

We suggest environmental and physiological changes are associated with increases or decreases in cancer risk. Vertebrate evolution has been associated with the adaptation to new environmental conditions, such as the invasion of land [4,7,8,9]. The increased metabolic rate in homeothermic and flying animals leads to a higher risk of mutation due to the accumulation of oxygen radicals, which requires the simultaneous development of an oncosuppressor mechanism [10,11]. Mammalian adaptations are also associated with increased cancer risk [12,13]. For example, the high body mass and lifespan of large, long-lived mammals promote somatic mutation accumulation due to an increase in cell quantity and lifespan (Peto’s paradox) [14]. Specific environmental adaptations in mammals, such as an underground lifestyle, cause changes in metabolic rate, the impact of cancerogenic xenobiotics, and cancer risk.

These adaptations may be associated with the development of various oncosuppressor mechanisms, which could be detected in the common ancestor of species exhibiting the trait or in all clades. Changes in the common ancestor may be associated with trait development. However, some changes in evolutionary rates may be related to trait development during further evolution and may be found in all clades with the trait [15].

An accelerated evolutionary rate may indicate positive or relaxed negative selection [16]. Oncosuppressor genes may undergo positive selection, which suggests the development of new antitumor protective mechanisms. Conversely, conservative oncosuppressor genes may undergo stabilizing selection to prevent harmful mutations in essential genes [17]. These hypotheses require further development and the implementation of a systems biology approach [18]. Tollis et al. used comparative genomics to identify positively selected tumor suppressor genes in large, long-lived mammals, providing a framework for our study [19].

An increase in cancer incidence in the human population is caused by environmental changes and an increase in lifespan [5,20,21]. However, evolutionary biology methods could help study the process of tumor development [22,23]. Therefore, studying the natural history of tumor suppressor genes can improve our understanding of cancer pathogenesis and pathomorphosis.

The aim of our study was to evaluate the strength and direction of selection acting on oncosuppressor genes associated with phenotypic changes, to improve our understanding of the molecular mechanisms underlying carcinogenesis.

## 2. Materials and Methods

### 2.1. Relative Evolutionary Rate

The Relative Evolutionary Rate (RER) method was employed to evaluate changes in the evolutionary rates of genes across phylogenetic trees. This method was originally developed to detect patterns of convergent evolution in mammals, and it was adapted for this study to evaluate the evolutionary pressures acting on oncosuppressor genes in a variety of vertebrates, including non-mammalian species [24,25]. The RER quantifies the divergence rate of specific gene sequences relative to a consensus phylogenetic tree. This enables the detection of selection pressures correlated with phenotypic traits, such as body mass, lifespan, and ecological adaptations.

The RER-based approach provides insights into the evolutionary dynamics of oncosuppressor genes and reveals potential adaptations linked to cancer risk across vertebrates. Acceleration in evolutionary rates of oncosuppressors often coincided with phenotypic adaptations such as increased body mass or lifespan, suggesting a balance between somatic maintenance and ecological fitness [26].

### 2.2. Genes and Species Datasets

All the species and genomic data were taken from UCSC database [27,28]. The aromorphosis analysis included 100 vertebrate species and 19,445 genes, including 154 of the most common oncosuppressor genes (Supplementary Table S2), selected from the COSMIC and UniProt databases [29,30]. The mammal trait analysis included 46 mammal species having 18 most common oncosuppressors in their genome. The total number of genes included was 7860.

The main types of oncosuppressor genes are cell cycle control genes (mostly associated with the G1/S checkpoint) and DNA repair genes. We analyzed selection pressure in DNA repair genes associated with the following processes: Base Excision Repair (BER), Nucleotide Excision Repair (NER), Homologous Recombination, Mismatch Excision Repair (MMR), non-homologous end-joining (NHEJ), Direct Reversal of Damage, conserved DNA damage response, DNA polymerases, editing and processing nucleases, Fanconi anemia pathway genes, and NER-related genes [31].

### 2.3. Trees Construction

**Figure 1:**
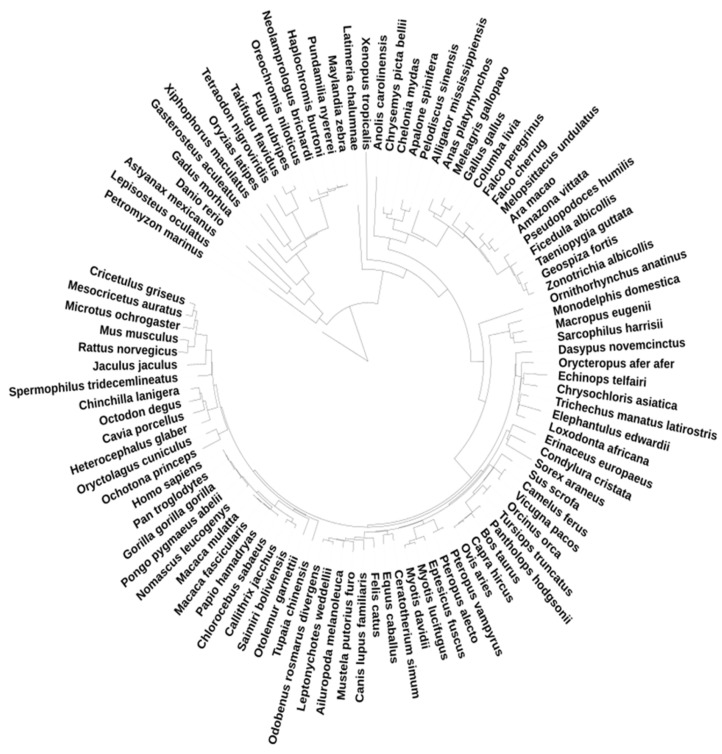
Consensus tree, the UCSC 100 vertebrates tree.

The amino acid sequences from the UCSC alignment were used [27], and the trees were constructed by the IQtree 2.0.6 tool with optimized model selection [32]. Given the discordance among numerous nodes in the Vertebrata tree, the constraints were utilized, employing those derived from vertebrate phylogeny as provided by the UCSC genome browser. We employed the UCSC tree as a consensus, built with the Maximum likelihood algorithm; the tree file was downloaded using the UCSC genome browser (Figure 1). The alignments were filtered: the species with more than 30% of gaps in the sequences were removed. All the manipulations with the alignments and trees were performed with Python 3.8 and the biopython module [28,33,34,35].

### 2.4. Traits

The analysis included traits associated with polygenic adaptation in mammals and vertebrates. We consider the included vertebrate traits as aromorphoses: flying adaptation, homeothermy, and amnion development [36].

To exclude the contribution of mammal and bird branches (75% of the tree), we conducted the amnion analysis, including only Reptilia (6 species) as foreground and Amphibia (1 species) and fishes (18 species) as background. All other vertebrate analyses included all the vertebrates from the UCSC genome browser; the mammalian analysis included 46 mammals. We considered birds and chiropterans as flying animals and studied them together and separately. Chiropteran analysis is presented in the “mammalian” part of the study.

The data on mammalian traits were obtained from the PanTHERIA database [37]. We divided the mammalian traits into “cancerogenic” traits, associated with various adaptations and control traits, possibly “non-cancerogenic”. Lifespan and body mass were suggested as the most promising parameters, potentially related to changes in oncosuppressor evolutionary rates. Therefore, we included several traits, associated with the parameters: continuous traits of lifespan and body mass, k-strategy and high body mass and lifespan (4 species)—4LL-HBM. K-strategy could be considered as an integrative parameter of mass and lifespan. K-strategy species are characterized by comparatively long lifespans and high body mass, which are required for effective adaptation to their ecological niches [38]. The continuous traits (body mass and lifespan) were log-normalized to approximate a normal distribution and reduce the influence of extreme values [39,40,41].

Other traits are associated with special mammal adaptations (Supplementary Table S1), such as high body mass and lifespan, K-strategy, underground adaptation, hibernation ability, sociality, and nocturnal lifestyle. We considered the four species having the highest body mass and the longest lifespan as having a special trait (4LL-HBM). A mammal body mass of more than 40 kg and a lifespan of more than 10 years were considered high [24,42,43,44].

### 2.5. RER Calculation

The RER method calculates the evolutionary rate of genes by comparing branch lengths from gene-specific phylogenetic trees to a consensus tree. Consensus tree branch lengths were computed as the average of all gene tree branch lengths. The RER is defined as the residual from a linear regression where the independent variable is the branch length from the consensus tree and the dependent variable is the branch length from the gene tree [25,26].

Variance-stabilizing transformations were applied to branch lengths to address heteroscedasticity and ensure accurate RER calculation across all vertebrates. The ancestral states of traits were reconstructed using the Maximum Parsimony Algorithm, and RERs were calculated for both terminal and internal branches. Foreground branches leading to species with specific traits were analyzed against all other background branches. To account for the phylogenetic signal, an independent analysis included both terminal branches and all internal nodes as foreground.

We calculated the RER, which measures the gene evolutionary rate in various species, and compared RERs for each gene in foreground and background branches. Foreground branches were initially selected: they led to species having a trait. All other branches were considered background. We included both internal and terminal branches in the analysis. The ancestral state was reconstructed with the Maximum Parsimony Algorithm, and only the internal branches were included in the analysis. We considered the most common ancestral branch as foreground and performed an independent analysis, including the terminal branches and all the internal nodes as foreground [24,25].

RERs are normalized genome-wide evolutionary rates, therefore the methods make it possible to minimize the impact of codon usage bias on evolutionary rate estimates. RER calculation helps control for systematic variation in substitution rates across branches.

### 2.6. Trait Analysis

The algorithm detects genes with significantly different RERs in foreground and background branches; Kendall correlation, *p*-value < 0.05, Benjamini–Hochberg correction. Foreground branches are considered to have the independent variable value “1”, which is “0” for background branches. We studied phenotypes as binary traits, presented only in foreground species.

The genes form two groups: “slow” and “fast” genes. “Fast” genes have significantly higher RER in foreground branches, and the evolutionary rate of the genes is accelerated in the foreground species; therefore, these genes could be characterized by positive or relaxed negative selection. In contrast, “slow” genes have significantly lower RER in foreground species. These genes have a decelerated evolutionary rate, being negatively selected [24,25].

In the case of the traits having continuous distribution, such as body mass or lifespan, we estimated the RER-trait correlation. For each gene, the dependent variable was a trait value and the independent variable was RER, Pearson correlation, *p*-value < 0.05, with Benjamini–Hochberg correction. Each species had a specific trait value and RER, included in the analysis. The correlation analysis was performed without the division of species into foreground and background.

We evaluated the list of the main oncosuppressors, being accelerated or decelerated. In addition, we employed the Fisher’s Exact Test to evaluate the probability of the oncosuppressor genes being accidentally included in the list of significantly correlated genes; *p*-value < 0.05. Fisher’s Exact Test was used as a secondary enrichment check rather than a primary hypothesis test. Because it was applied post hoc to verify overrepresentation, we did not apply False Discovery Rate (FDR) correction.

### 2.7. Enrichment Analysis

We performed enrichment analysis to detect functional groups of genes significantly associated with a trait. Various genes from the same group may have different signs of the correlation coefficient. Therefore, the foreground acceleration rate (FAS) was calculated for each gene (Partha et al., 2019) [25]: FAS = rho(sign)*[−log(p)], where FAS—foreground acceleration rate; rho (sign) is the sign of the correlation coefficient; p is the *p*-value; and stat is the foreground acceleration rate.

The Foreground Acceleration Rate (FAS) integrates the magnitude and direction of evolutionary rate changes (correlation coefficient) with statistical significance (−log(*p*-value)). Gene sets are annotated using MSigDb Hallmarks and Canonical pathways, and their enrichment is assessed via the Mann–Whitney test; *p*-value < 0.05, FDR.

Hallmarks are gene sets aggregating many MSigDB gene sets to represent well-defined biological states or processes (total number 50). Canonical pathways are gene sets from pathway databases; the total number is 2982 [45,46]. Using MSigDb Hallmarks and Canonical pathways, we performed enrichment analysis to identify functional pathways associated with traits. Significantly enriched Canonical pathways were clustered using the Jaccard similarity index (J > 0.5), enabling the identification of higher-order biological processes.

## 3. Results

### 3.1. Vertebrate Trait Analysis

**Figure 2:**
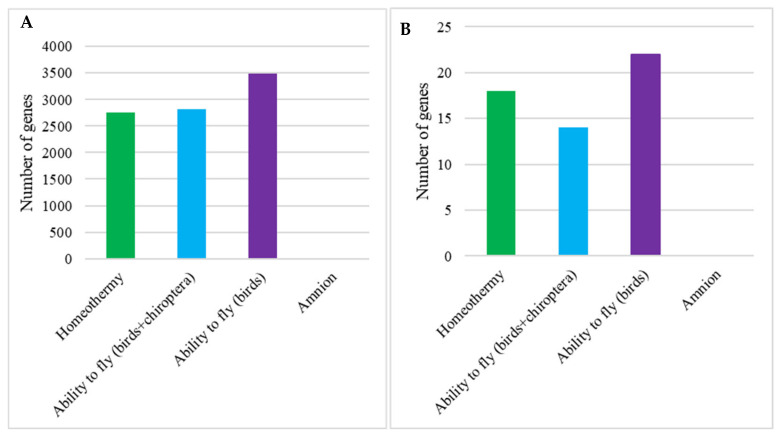
Single genes correlated with the aromorphoses. The genes are significantly correlated with the trait (RER-trait correlation, Kendall method; *p*-value < 0.05 with Benjamini–Hochberg correction). (**A**) Number of all the genes significantly correlated with the trait. (**B**) Number of oncosuppressor genes significantly correlated with the trait. The number of genes is shown at the ordinate. There were no significantly correlated genes in amniotic species.

The study did not detect genes with significantly correlated RER increase/decrease in the common ancestors of the homeothermic, amniotic, and flying species. However, studying all the branches, ancestral and terminal, we detected genes that significantly correlated with homeothermy and the ability to fly; meanwhile, there were no genes significantly correlated with amnion development (Figure 2). There was no difference between observed and expected oncosuppressor incidence in the group of significantly correlated genes.

**Figure 3:**
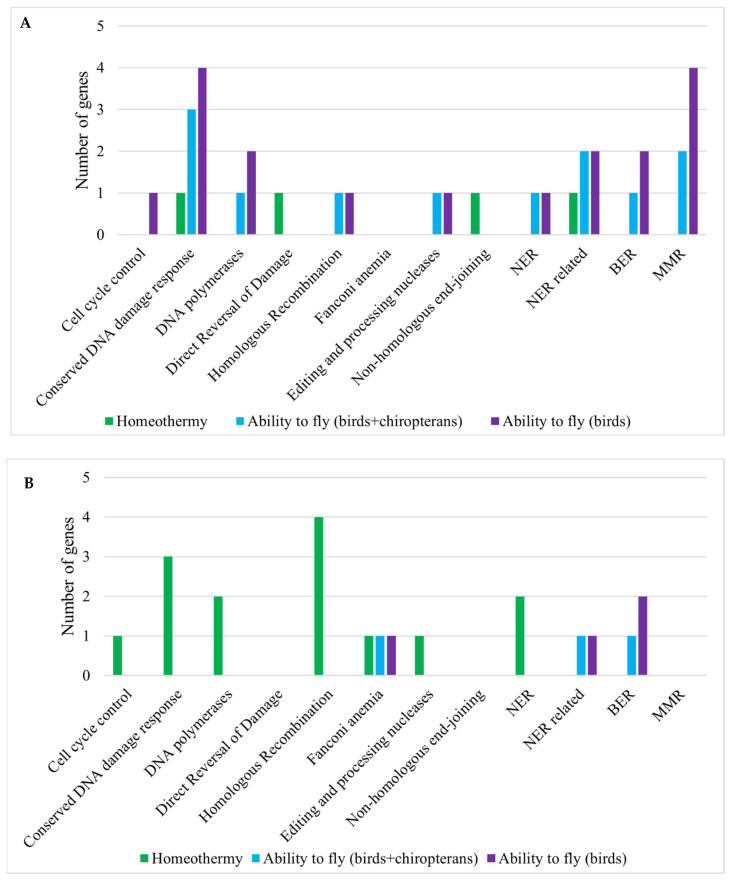
Main oncosuppressor genes significantly correlated with vertebrate aromorphoses. The numbers of genes in each oncosuppressor class are presented (Section 2.2). The genes are significantly correlated with the trait (RER-trait correlation, Kendall method, *p*-value < 0.05 with Benjamini–Hochberg correction). The gene numbers are presented on the ordinate. (**A**) Accelerated genes; (**B**) Decelerated genes.

We detected various classes of oncosuppressors positively and negatively correlated with the traits. In flying animals (birds and chiropterans), 78.6% of the genes were positively correlated with the trait; Fisher’s Exact Test *p*-value = 0.03. The genes from 11 out of 13 oncosuppressor classes were detected. The genes were equally spread between the classes (Figure 3); Fisher’s Exact Test *p*-value = 0.65. The same results were obtained in bird branches: 82% of the genes were accelerated, Fisher’s Exact Test *p*-value = 0.02; and genes were equally spread between the classes; *p*-value = 0.68.

In the case of homeothermy, most of the significantly correlated oncosuppressors were decelerated in association with the trait (77.8%, *p*-value = 0.04), which may indicate stabilizing selection (Figure 3). The genes from 9 out of 13 oncosuppressor classes were detected. The genes were equally spread between the groups; *p*-value = 0.54.

The results of our vertebrate individual genes analysis should be interpreted with caution, due to the results of Fisher’s Exact Test (Figure 2 and Figure 3). Oncosupressors, significantly correlated with the traits in all the branches, are expected to be accelerated in adapting to different ecological niches. In some populations, having a trait may result in adapting to new environmental conditions, which leads to speciation. This process may require some genes to be under positive or stabilizing selection.

The significantly correlated genes (without Benjamini–Hochberg correction) were included in the enrichment analysis. We annotated the genes with the molecular signature database (MSigDb). We detected two types of enrichment: enriched hallmark pathways, associated with the most common cell molecular processes (all the genes were divided into 50 hallmarks), and Canonical pathways, which are more specific. We considered the average gene Rho a rate of pathway acceleration/deceleration.

**Figure 4:**
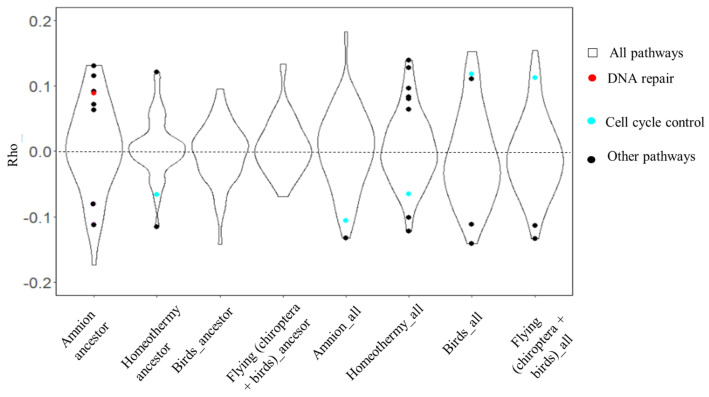
Hallmark enrichment analysis for vertebrate traits. Points are hallmark pathways, significantly enriched for significantly correlated genes; *p*-value < 0.05, Benjamini–Hochberg correction. Violins illustrate all the hallmarks presented in the species. DNA repair pathways are red, cell cycle control pathways are blue, and other pathways are black. Average Rho for each pathway is presented on the ordinate. Traits are presented on the abscissa.

The analysis detected significantly enriched hallmarks, associated with cell antitumor mechanisms in association with all the aromorphoses (Figure 4). In flying animals, no hallmark pathways were significantly enriched in their ancestral branches. However, analyzing all the branches, the G2/M checkpoint pathway was accelerated, similarly to homeothermic species. Interestingly, this trend persisted when birds were analyzed independently from chiropterans, suggesting convergent evolution in response to similar metabolic and cellular pressures.

**Figure 5:**
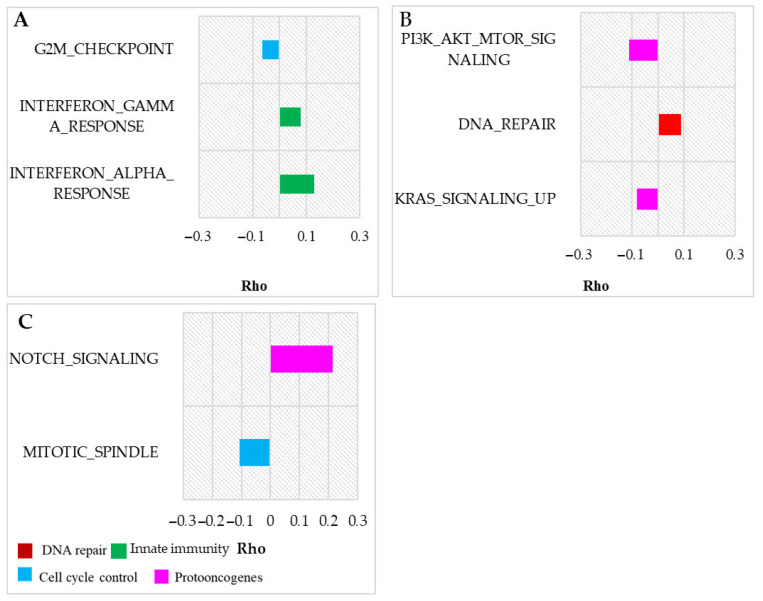
Anti-oncogenic and cancer-related hallmark pathways significantly enriched in vertebrate trait analysis. (**A**) Homeothermic species—all branches. (**B**) Amniotic species—only common ancestor branches. (**C**) Amniotic species—all branches. Here are presented hallmark pathways, significantly enriched for significantly correlated genes in all the branches; *p*-value < 0.05, Benjamini–Hochberg correction. Average gene Rho (Kendall RER-trait correlation) for each pathway is presented on the abscise.

Genes significantly negatively correlated with homeothermy were significantly enriched for the G2/M checkpoint hallmark (Figure 5). The G2/M checkpoint is a pathway involved in cell cycle regulation, responsible for the apoptosis of cells with damaged DNA, preventing mutated cells on the G2 stage from mitosis. This pathway was decelerated in homeothermic animals in the ancestor branches and all the branches. The detected negative trait-pathway correlation indicates the stabilizing selection of the genes, enriched for the G2/M checkpoint pathway.

Innate immunity pathways were accelerated in all the branches of homeothermic species (Figure 5A). Therefore, these pathways are possibly under positive/relaxed negative selection. The immune system is involved in anti-tumoral mechanisms. Interferon-gamma is associated with innate immunity; it regulates CD4+ T-cell function and modulates antitumoral antibody production. Additionally, interferon-gamma controls CD8+ cell function, which is crucial for tumor growth control, inducing malignant cell apoptosis. Interferon-alpha is also involved in antitumoral and antiviral protective reactions [47,48,49,50].

For the amnion trait, DNA repair genes were accelerated in the ancestral branches. However, the MSigDb Hallmark DNA repair pathway did not exhibit acceleration across all branches (Figure 5B,C). This discrepancy may indicate that the increase in evolutionary rate is confined to the ancestral branch, highlighting the specificity of trait–pathway associations in early evolutionary contexts.

Our analysis across all branches identified the Mitotic Spindle pathway, associated with cell cycle control, as being decelerated in association with the trait (Figure 5B). The results show changes in gene evolutionary rates in reptiles compared to fishes; we only included reptiles as amniotic species to decrease the contribution of mammalian and bird branches. In addition, UCSC alignments include only one species of Amphibia, so its contribution should be neglected.

Proto-oncogenic pathways are associated with various cell functions and cancer protection. Mutations cause abnormal activation of the biochemical cascades or destroy them, which leads to malignancy. Some pathways such as KRAS, PI3AKT, and mTOR are pathologically activated. mTOR pathways (MTORC1 and PI3AKT MTOR) include insulin-growth factor metabolism. KRAS is a GTPase, and its activation changes the expression of growth factors and their receptors that cause malignant transformation [51]. Mutations in highly conservative cell–cell transduction pathways (Notch and Wnt pathways) are often associated with carcinogenesis.

Some of the pathways were decelerated (Notch, Wnt), which indicates negative selection. However, the acceleration of RER in proto-oncogenic pathways in association with the trait requires further investigation. Generally, the results may indicate relaxed negative or positive selection in accelerated pathways. mTOR and Notch are involved in adaptive immunity development and T-cell differentiation, which explains their positive selection [47]. The adaptive immune system may evolve in response to cancer risk increase in mammal and homeothermic vertebrates [52], which have a high cancer risk due to an increase in the metabolic rate.

**Figure 6:**
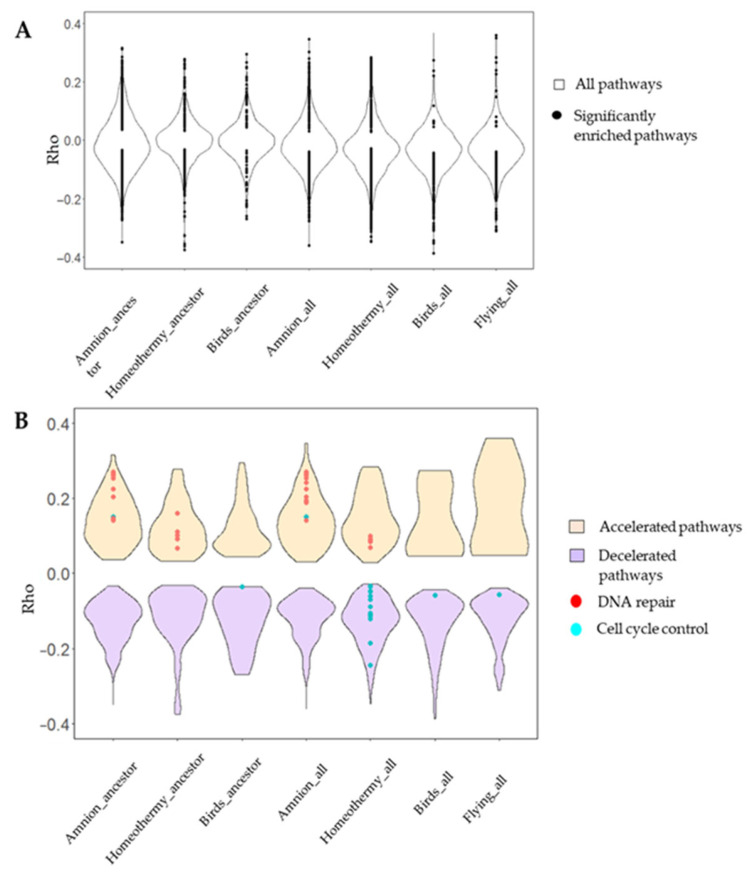
Canonical pathways associated with vertebrate traits: (**A**) Rho for all the pathways (violin plots). Black points indicate significantly enriched pathways. (**B**) Rho for oncosuppressor pathways. Accelerated pathways are orange, and decelerated pathways are purple (violin plots). DNA repair (red) and cell cycle control (cyan) pathways are presented for each trait. Average gene Rhos were calculated for each pathway based on Kendall RER–trait correlation and presented on the ordinate.

Canonical pathways enrichment analysis detected the pathways significantly accelerated and decelerated in association with the traits (Figure 6A). We did not detect any canonical pathways being presented both in ancestor branches of chiropterans and birds—flying animals. This finding is associated with a lack of common genes for the branches. Initially, any ancestor branch may have fewer genes than all the terminal branches: only common genes are presented in the ancestor. At the same time, the ancestors of chiropterans and birds are far from each other on the tree; therefore, they do not have enough genes with the same functions to be included in a canonical pathway (the borderline was 10). However, hallmarks consist of larger pathways, including hundreds of genes, so the enrichment analysis was successfully performed.

We detected significantly accelerated DNA repair genes in the ancestors of homeothermic species without significant acceleration in all branches (Figure 6B). We also detected cell cycle control genes being significantly decelerated in all the branches, but some pathways were accelerated as well. In flying animals, only one pathway was significantly enriched in birds and all flying animals, with an average gene Rho below zero. In amniotic species, we detected significantly enriched DNA repair and cell cycle control pathways that were mostly accelerated in association with the common ancestor and all branches.

### 3.2. Mammal Trait Analysis

Our analysis revealed individual genes with accelerated or decelerated evolutionary rates associated with continuous traits such as lifespan and body mass, as well as with K-strategy (ancestral branches). However, no specific oncosuppressor genes showed significant correlations across all traits.

**Figure 7:**
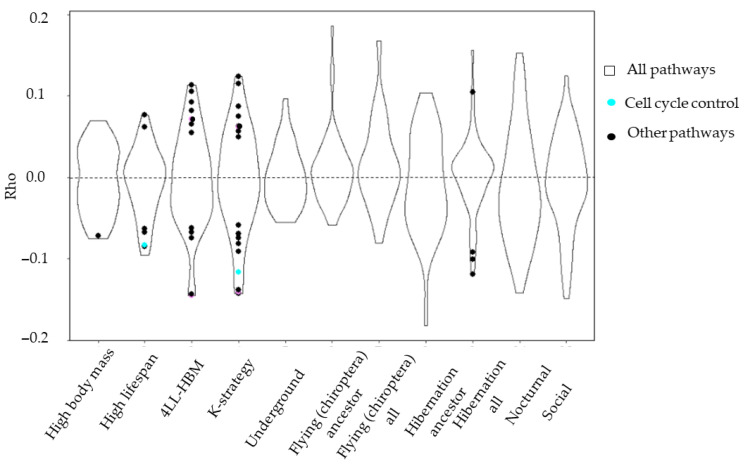
Hallmark enrichment analysis for mammal traits. Points are hallmark pathways significantly enriched for significantly correlated genes (*p*-value < 0.05, Benjamini–Hochberg correction.) Violins illustrate all hallmarks presented in the species. Cell cycle control pathways are blue, other pathways are black. Average gene Rhos were calculated for each pathway based on Kendall RER–trait correlation and presented on the ordinate.

Although oncosuppressor hallmarks were not significantly enriched in association with an increase in body mass (Figure 7), the G2/M checkpoint pathway exhibited significant enrichment and deceleration in correlation with lifespan. The G2/M checkpoint pathway also exhibited deceleration in K-strategy species. No hallmarks were significantly enriched in branches associated with environmental and metabolic adaptations, such as underground lifestyles and hibernation. Similarly, traits such as sociality and nocturnal lifestyles, initially considered non-cancer-related, did not exhibit significant hallmark enrichment. These traits have evolved independently across various species and lack a constitutive genetic basis. Our findings suggest that their development is not associated with shifts in cancer risk or oncosuppressor evolutionary rates.

**Figure 8:**
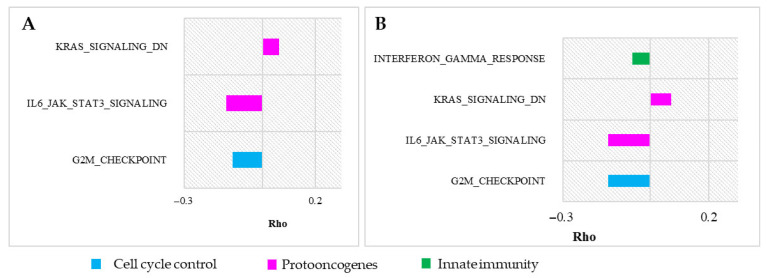
Anti-oncogenic and cancer-related hallmark pathways significantly enriched in association with body mass and lifespan changes. Hallmark pathways significantly enriched for significantly correlated genes are presented; *p*-value < 0.05, Benjamini–Hochberg correction. Average gene Rhos (Kendall RER–trait correlation) for each pathway are presented on the abscissa. Cell cycle control pathways are blue, pro-oncogenic pathways are purple, and innate immunity pathways are green. (**A**) K-strategy species; (**B**) Lifespan (continuous trait).

Furthermore, we identified a decelerated innate immunity pathway, specifically interferon-gamma, in the four long-lived, high-body mass species (4LL-HBM), suggesting a potential link to antitumor immunity [48,49,50]. Similar findings were reported by Kowalczyk et al. in their investigation of mammalian antitumoral mechanisms in relation to lifespan and body mass [24]. These results collectively indicate stabilizing selection acting on these pathways (Figure 8).

**Figure 9:**
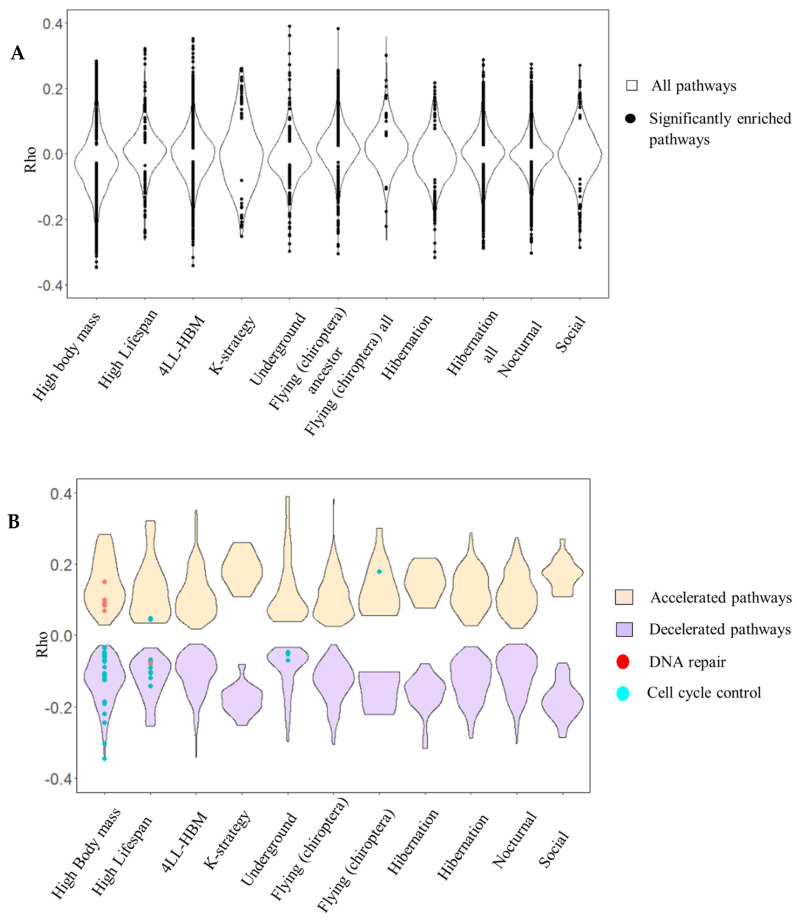
Canonical pathways associated with mammal traits. (**A**) Rho for all the pathways (violin plots). Black points indicate significantly enriched pathways. (**B**) Rho for oncosuppressor pathways. Accelerated pathways are orange and decelerated pathways are purple (violin plots). DNA repair (red) and cell cycle control (cyan) pathways are presented for each trait. Average gene Rhos were calculated for each pathway based on Kendall RER–trait correlation and presented on the ordinate.

Although no hallmarks showed similar enrichment, canonical pathways related to cell cycle control were significantly enriched in association with underground adaptation (Figure 9A). These pathways include smaller, more specific gene sets than hallmarks, which may explain the discrepancy. No significant enrichment was detected for either canonical pathways or hallmarks for other mammalian adaptations, such as social and nocturnal lifestyles, flying, and hibernation.

Our analysis revealed divergent patterns for traits such as lifespan and body mass. DNA repair pathways decelerate in association with increases in lifespan, while accelerating in relation to increases in body mass (Figure 9B). In contrast, cell cycle control pathways are consistently decelerated in relation to both traits. The findings suggest that hallmark enrichment reflects broader evolutionary rate shifts across larger gene sets, whereas canonical pathway enrichment often captures more specific adaptations within smaller gene subsets. This distinction likely explains the results observed for K-strategy species, in which small gene group deceleration was evident in canonical pathways, but not in overlapping hallmark pathways, after FDR correction.

## 4. Discussion

Our study examined the relationship between trait selection in vertebrates and mammals and changes in the evolutionary rates of oncosuppressor genes. Our analysis revealed key patterns of evolutionary pressures acting on DNA repair and cell cycle control genes across phylogenetic lineages. These results support the hypothesis that traits linked to metabolic rate changes and environmental adaptation are associated with shifts in antitumor gene selection.

### 4.1. Evolutionary Patterns in Oncosuppressor Genes

We estimated the strength and direction of antitumoral gene selection in various vertebrates and mammals and studied the association between changes in oncosuppressor evolutionary rates and trait selection. We examined changes in evolutionary rates among species with a trait and their most common ancestors.

We examined how changes in oncosuppressor evolutionary rates were associated with vertebrate and mammalian trait selection. Common vertebrate traits are considered crucial and sufficient adaptations associated with environmental changes and metabolic adaptations to new ecological niches, i.e., aromorphoses [36]. Meanwhile, adaptations within the mammal class are smaller but are all associated with polygenic environmental adaptation [53,54].

Oncosuppressor genes, typically classified as housekeeping genes, are highly conserved due to their crucial roles in cell cycle regulation and DNA repair [55]. We observed that individual oncosuppressor genes showed accelerated evolutionary rates in association with flight adaptations and decelerated rates in homeothermic species. These patterns suggest positive or relaxed negative selection in the former and stabilizing selection in the latter. DNA repair genes exhibited acceleration in ancestral branches of amniotes and homeothermic clades, reflecting their role in addressing increased oxidative stress. Conversely, cell cycle control genes, particularly those involved in the G2/M checkpoint pathway, were consistently decelerated across homeothermic animal clades, indicating purifying selection (Figure 3 and Figure 5A).

Our findings support the hypothesis that the metabolic rate increases associated with homeothermy and flight drive the evolution of antitumor mechanisms. Chiari et al. presented the same suggestion [56]. Homeothermy and flying adaptations lead to a significant increase in metabolic rate [57]. A stable high body temperature is a suggested risk factor for cancer due to its association with increased metabolic rate [18]. Therefore, birds have a high cancer risk due to homeothermy and their ability to fly, necessitating additional antitumoral protection. Our results support this hypothesis; we detected stabilizing selection of crucial ancient cell cycle control genes and pathways, as well as positive and relaxed negative selection of additional DNA repair genes.

We only included reptiles, amphibians, and fish in the amnion trait analysis, which make up 25% of the tree. Nevertheless, we detected some decelerated pathways in association with the trait (all branches). Compared to fish, reptiles, as amniotic species, have an increased cancer risk due to higher oxidative stress. A terrestrial lifestyle is associated with higher insolation and xenobiotic impact [18,19,58].

All flying vertebrates in our dataset (bats and birds) are homeothermic and have decelerated cell cycle control pathways. These traits are associated with an increased metabolic rate and cancer risk; therefore, we acknowledge the potential confounding factors between these traits. However, our trait-specific analyses (e.g., separate analyses of birds and bats) revealed differences in the evolutionary rates of single genes and pathways that indicate convergence between different taxonomic groups (Figure 2 and Figure 3).

Our results indicate that the acquisition of major traits (aromorphoses) like flight and homeothermy is associated with significant shifts in the evolutionary rates of oncosuppressor genes. The observed acceleration of these genes in flying lineages (suggesting positive or relaxed selection) and their deceleration in homeotherms (suggesting stabilizing selection) aligns with the well-established evolutionary concept that populations adapting to new niches experience selective pressures that can lead to speciation. This process often requires core cellular functions to be stabilized (stabilizing selection) while simultaneously allowing for innovation and adaptation in other genes (positive selection) to meet new environmental challenges [16,56]. Therefore, our results indicate the crucial role of oncosuppressors selection in the course of evolution.

### 4.2. Insights into Mammalian-Specific Adaptations

Our analysis did not identify any oncosuppressor genes that were significantly accelerated or decelerated in association with mammalian trait selection. However, we observed accelerated DNA repair gene expression in association with increased body mass in mammals (positive/relaxed negative selection) (Figure 5B and Figure 6). Conversely, cell cycle control genes were decelerated in long-lived, high-body-mass, and underground mammals (stabilizing selection) (Figure 7). Notably, no significant acceleration or deceleration of oncosuppressor pathways was detected in association with hibernation. High lifespan and body mass are considered the main adaptations in mammals and are associated with an increased cancer risk due to cell mass and somatic mutation accumulation. Increases in body mass and lifespan are associated with the development of additional oncosuppressor mechanisms [19,43,59].

Continuous traits, such as body mass and lifespan, exhibit constitutive acceleration and deceleration within genes. These traits are calculated without considering branch division in the foreground or background; all internal and terminal branches are assumed to exhibit the same trait manifestations. Thus, we propose that cell cycle control genes, particularly the G2/M checkpoint, enable these traits. Stabilizing selection prevents the loss of oncosuppression mechanisms. There is a connection between the evolution of oncosuppression and body mass. Kowalczyk et al. identified genes associated with trait development (the ancestral state of the most long-lived and body-mass species) and trait enablement [24]. In our study, we included a restricted list of mammals with the most important oncosuppressors in their genomes and examined the genes present in all species with the trait. We did not detect pathways significantly enriched in the ancestors of species with high body mass and longevity. An increase in the amount of oncosuppressors per genome could be an independent mechanism of anti-tumoral protection. Meanwhile, in the case of the 4LL-HMB species (binary trait), we compared RERs in the background and foreground branches. Including species with more oncosuppressors may allow us to compare the evolutionary rate of oncosuppressors between species that were initially more protected.

Similar results were obtained in studies dedicated to underground species. A genome-wide association study detected wide anti-tumoral adaptations, including DNA-repair mechanisms [60,61]. Specific antitumor adaptations were identified in naked mole rats, which have an average lifespan of about 32 years. Therefore, these findings may be associated with adaptations to a long lifespan. However, Jiang et al. detected positive selection in the DNA repair and adaptive immune pathways of *Condylura cristata*, *Chrysochloris asiatica*, and *Fukomys damarensis* [61]. We detected only a few cell cycle control pathways under stabilizing selection. At the same time, Jiang et al. only compared a small group of rodents. Our study included two species, *Condylura cristata* and *Chrysochloris asiatica* (foreground), and compared them with 44 other mammal species (background). We observed deceleration in innate immunity pathways (e.g., interferon-gamma signaling), which aligns with previous findings by Kowalczyk et al. These immune functions also contribute to tumor suppression and appear to be under stabilizing selection [47,48,49,50].

In flying animals, individual genes show acceleration, but pathway-level enrichment is less pronounced, suggesting that flight adaptation may involve subtle, distributed changes across many oncosuppressor genes rather than strong, coordinated shifts in entire functional pathways. At the same time, the G2/M checkpoint pathway was accelerated across all branches. Hibernation was not associated with changes in the evolutionary rates of oncosuppressors. However, it is possible that hibernation is associated with changes in cancer risk due to changes in metabolic rate and oxygen radical generation. Conversely, the metabolic rate decreases during hibernation, which is associated with a decreased cancer risk [62].

We did not detect significant enrichment of oncosuppressor pathways for other mammalian traits, such as hibernation, sociality, and nocturnal lifestyles (Figure 6 and Figure 8). These traits evolved independently in different species and likely do not involve consistent shifts in cancer risk or oncosuppressor selection. Complex models integrating phenotypic and genotypic evolution may be necessary to understand these patterns fully [63,64]. Thus, our study confirmed that the selection of traits possibly not associated with significant environmental changes or changes in metabolic rate does not lead to the selection of oncosuppressors.

DNA repair genes have different functions and are relatively variable. They are positively selected in association with trait development. Cell cycle control genes are more conservative and are under stabilizing selection [18,19]. Thus, the evolutionary pressure to maintain proto-oncogene function is balanced by the risk of oncogenic mutations. Innate immunity genes undergo rapid evolution due to the continuous battle between hosts and pathogens. Positive selection is often seen in these genes, leading to the diversification of immune receptors and the growth of gene families that recognize and respond to pathogens [65,66]. Proto-oncogenes demonstrate equilibrium between cellular growth and cancer risk, while innate immunity genes exemplify the perpetual struggle between hosts and pathogens.

### 4.3. Study Novelty and Limitations

Our results reveal an association between trait selection and changes in the evolutionary rates of oncosuppressors in vertebrate aromorphoses and mammalian adaptations. First, we detected differences in the evolutionary rates of cell cycle control and DNA repair genes under trait selection pressure. One limitation of the study is the small species list: 100 vertebrates and 46 mammals. For this reason, we were unable to evaluate amphibian and reptile adaptations. Reptiles, amphibians, and fish comprise only 25% of the species presented on the UCSC tree.

## 5. Conclusions

Our findings demonstrate that the G2/M checkpoint pathway decelerates in association with vertebrate traits, such as homeothermy, and with mammalian adaptations, such as increased body mass and lifespan. These findings suggest purifying selection. This pathway is involved in cell cycle control, induces apoptosis, and prevents the transition from the G2 phase to mitosis in the event of DNA damage. Aromorphoses and body mass/lifespan adaptations are the most “carcinogenic.” Our study revealed the critical role of the G2/M checkpoint pathway in developing and enabling these traits. Furthermore, vertebrate and mammalian adaptations are associated with changes in the evolutionary rate of oncosuppressors, including innate immunity genes, which suggest a broader spectrum of adaptive mechanisms warranting further exploration. Thus, our findings provide evolutionary insight into the molecular strategies vertebrates and mammals have developed to counteract cancer risk. They also highlight the importance of integrating trait-specific evolutionary signals with systems-level analyses of tumor suppression mechanisms.

## Data Availability

The original contributions presented in this study are included in the article and Supplementary Materials. Further inquiries can be directed to the corresponding author.

## Supplementary Materials

The following supporting information can be downloaded at https://www.mdpi.com/article/10.3390/life15101556/s1, Table S1: Mammal trait-species data; Table S2: Main oncosuppressors, included in individual gene analysis.

## Abbreviations

The following abbreviations are used in this manuscript:

DNA	Deoxyribonucleic Acid
RER	Relative Evolutionary Rate
UCSC	University of California Santa Cruz
