# Supplementary material for: Evolutionary Dynamics of Oncosuppression Under Selection Pressure
Source: Life (Basel). 2025 Oct 3;15(10):1556. doi: 10.3390/life15101556 (PMC12565122; doi:10.3390/life15101556)
Supplement: Supplementary file 1 [file life-15-01556-s001.zip › Table S2.pdf]

Table S2. Main oncosupressors, included in individual gene analysis.

Gene	Related pathway	Gene	Related pathway	Gene	Related pathway
APEX1	Base Excision Repair (BER)	TP53	Conserved DNA damage response	TREX2	Editing and processing nucleases
APEX2	Base Excision Repair (BER)	MDC1	Conserved DNA damage response	FAN1	Editing and processing nucleases
LIG3	Base Excision Repair (BER)	TOPBP1	Conserved DNA damage response	APTX	Editing and processing nucleases
MBD4	Base Excision Repair (BER)	CLK2	Conserved DNA damage response	FANCA	Fanconi anemia
MPG	Base Excision Repair (BER)	PER1	Conserved DNA damage response	FANCE	Fanconi anemia
MUTYH	Base Excision Repair (BER)	TP53BP1	Conserved DNA damage response	FANCB	Fanconi anemia
NEIL1	Base Excision Repair (BER)	RIF1	Conserved DNA damage response	FANCC	Fanconi anemia
NEIL2	Base Excision Repair (BER)	ALKBH3	Direct Reversal of Damage	FANCD2	Fanconi anemia
NEIL3	Base Excision Repair (BER)	ALKBH2	Direct Reversal of Damage	FANCF	Fanconi anemia
NTHL1	Base Excision Repair (BER)	MGMT	Direct Reversal of Damage	FANCG	Fanconi anemia
OGG1	Base Excision Repair (BER)	MAD2L2	DNA polymerases	FANCI	Fanconi anemia
PARG	Base Excision Repair (BER)	PCNA	DNA polymerases	FANCL	Fanconi anemia
PARP1	Base Excision Repair (BER)	POLA1	DNA polymerases	FANCM	Fanconi anemia
PARP2	Base Excision Repair (BER)	POLB	DNA polymerases	SLX4	Fanconi anemia
PARP3	Base Excision Repair (BER)	POLD1	DNA polymerases	BRIP1	Fanconi anemia
PNKP	Base Excision Repair (BER)	POLE	DNA polymerases	PALB2	Fanconi anemia
SMUG1	Base Excision Repair (BER)	POLG	DNA polymerases	BRCA1	Homologous Recombination
TDG	Base Excision Repair (BER)	POLH	DNA polymerases	BRCA2	Homologous Recombination
UNG	Base Excision Repair (BER)	POLI	DNA polymerases	DMC1	Homologous Recombination
XRCC1	Base Excision Repair (BER)	POLK	DNA polymerases	EME1	Homologous Recombination
ATR	Conserved DNA damage response	POLL	DNA polymerases	EME2	Homologous Recombination
ATRIP	Conserved DNA damage response	POLM	DNA polymerases	GEN1	Homologous Recombination

CHEK1	Conserved DNA damage response	POLN	DNA polymerases	MUS81	Homologous Recombination
CHEK2	Conserved DNA damage response	POLQ	DNA polymerases	NBN	Homologous Recombination
HUS1	Conserved DNA damage response	REV1	DNA polymerases	RAD21	Homologous Recombination
RAD1	Conserved DNA damage response	REV3L	DNA polymerases	RAD50	Homologous Recombination
RAD17	Conserved DNA damage response	EXO1	Editing and processing nucleases	RAD51	Homologous Recombination
RAD9A	Conserved DNA damage response	FEN1	Editing and processing nucleases	RAD51C	Homologous Recombination
RRM1	Conserved DNA damage response	ENDOV	Editing and processing nucleases	RAD51B	Homologous Recombination
RRM2	Conserved DNA damage response	SPO11	Editing and processing nucleases	RAD51D	Homologous Recombination
RRM2B	Conserved DNA damage response	TREX1	Editing and processing nucleases	RAD52	Homologous Recombination
RAD54B	Homologous Recombination	SMC6	Homologous Recombination	LIG1	Nucleotide Excision Repair (NER)
RAD54L	Homologous Recombination	XRCC2	Homologous Recombination	MNAT1	Nucleotide Excision Repair (NER)
SMC6	Homologous Recombination	XRCC3	Homologous Recombination	RAD23A	Nucleotide Excision Repair (NER)
XRCC2	Homologous Recombination	RBBP8	Homologous Recombination	RAD23B	Nucleotide Excision Repair (NER)
XRCC3	Homologous Recombination	SLX1A	Homologous Recombination	RPA1	Nucleotide Excision Repair (NER)
RBBP8	Homologous Recombination	SLX1B	Homologous Recombination	RPA2	Nucleotide Excision Repair (NER)
SLX1A	Homologous Recombination	MLH1	Mismatch Excision repair (MMR)	RPA3	Nucleotide Excision Repair (NER)
SLX1B	Homologous Recombination	MLH3	Mismatch Excision repair (MMR)	XPA	Nucleotide Excision Repair (NER)

MLH1	Mismatch Excision repair (MMR)	MSH2	Mismatch Excision repair (MMR)	XPC	Nucleotide Excision Repair (NER)
MLH3	Mismatch Excision repair (MMR)	MSH3	Mismatch Excision repair (MMR)	ERCC8	NER related
MSH2	Mismatch Excision repair (MMR)	MSH4	Mismatch Excision repair (MMR)	DDB1	NER related
MSH3	Mismatch Excision repair (MMR)	MSH5	Mismatch Excision repair (MMR)	DDB2	NER related
MSH4	Mismatch Excision repair (MMR)	MSH6	Mismatch Excision repair (MMR)	ERCC6	NER related
MSH5	Mismatch Excision repair (MMR)	PMS1	Mismatch Excision repair (MMR)	MMS19	NER related
MSH6	Mismatch Excision repair (MMR)	PMS2	Mismatch Excision repair (MMR)	UVSSA	NER related
PMS1	Mismatch Excision repair (MMR)	DCLRE1C	Non-homologous end-joining	XAB2	NER related
PMS2	Mismatch Excision repair (MMR)	LIG4	Non-homologous end-joining	TP53	Conserved DNA damage response
DCLRE1C	Non-homologous end-joining	PRKDC	Non-homologous end-joining	TP63	Cell cycle control
LIG4	Non-homologous end-joining	NHEJ1	Non-homologous end-joining	TP73	Cell cycle control
PRKDC	Non-homologous end-joining	XRCC4	Non-homologous end-joining	RB1	Cell cycle control
NHEJ1	Non-homologous end-joining	XRCC5	Non-homologous end-joining	PTEN	Cell cycle control
XRCC4	Non-homologous end-joining	XRCC6	Non-homologous end-joining	CDKN2B	Cell cycle control
XRCC5	Non-homologous end-joining	CCNH	Nucleotide Excision Repair (NER)	CDKN2C	Cell cycle control
XRCC6	Non-homologous end-joining	CDK7	Nucleotide Excision Repair (NER)	CDKN2D	Cell cycle control
CCNH	Nucleotide Excision Repair (NER)	ERCC1	Nucleotide Excision Repair (NER)	CDKN1C	Cell cycle control

CDK7	Nucleotide Excision Repair (NER)	ERCC2	Nucleotide Excision Repair (NER)	CCND1	Cell cycle control
ERCC1	Nucleotide Excision Repair (NER)	ERCC3	Nucleotide Excision Repair (NER)	CCNE2	Cell cycle control
ERCC2	Nucleotide Excision Repair (NER)	ERCC4	Nucleotide Excision Repair (NER)		
ERCC3	Nucleotide Excision Repair (NER)	FBXL2	Nucleotide Excision Repair (NER)		
ERCC4	Nucleotide Excision Repair (NER)	GTF2H1	Nucleotide Excision Repair (NER)		
FBXL2	Nucleotide Excision Repair (NER)	GTF2H2	Nucleotide Excision Repair (NER)		
RAD54L	Homologous Recombination	GTF2H3	Nucleotide Excision Repair (NER)		
