# Supplementary material for: Evolutionary Dynamics of Oncosuppression Under Selection Pressure
Source: Life (Basel). 2025 Oct 3;15(10):1556. doi: 10.3390/life15101556 (PMC12565122; doi:10.3390/life15101556)
Supplement: Supplementary file 1 [file life-15-01556-s001.zip › Table S1.pdf]

Table S1. Mammal trait-species data.

Species \ Traits	Hibernation	Underground	social	K-strategy	nocturnal	Average lifespan, years	Average body mass, g
**Monodelphis domestica**					+	4	122,5
**Orycteropus afer**				+	+	18	61000
**Echinops telfairi**	+				+	2,7	265
**Chrysochloris asiatica**	+	+			+	no information	42
**Trichechus manatus**				+		30	400000
**Elephantulus edwardii**					+	5,8	50
**Loxodonta africana**				+		60	4050000
**Tupaia chinensis**						10,5	160
**Otolemur garnettii**					+	14	771,5
**Callithrix jacchus**			+	+		10	320
**Nomascus leucogenys**			+	+		28	5700
**Pan troglodytes**			+	+		51	48000
**Homo sapiens**			+	+		60	70000
**Chlorocebuss abaeus**			+	+		12	5750
**Papio hamadryas**			+	+		37	15250
**Macaca fascicularis**			+	+		37	5000
**Ochotona princeps**			+			3	148,5
**Heterocephalus glaber**		+	+			15	33,5
**Cavia porcellus**			+			8	900
**Octodon degus**			+			6,5	235
**Chinchilla lanigera**			+		+	10	650
**Spermophilus tridecemlineatus**	+		+			3	125
**Criceulus griseus**	+		+			2,5	37
**Microtus ochrogaster**			+			1	50
**Rattus norvegicus**			+		+	2	400
**Mus musculus**			+		+	1,25	21
**Erinaceus europaeus**	+				+	6	1000
**Condylura cristata**		+	+			3,5	55
**Sorex araneus**					+	2	19,5

**Camelus ferus**			+	+			30	495000
**Vicugna pacos**			+	+			7,5	60000
**Orcicus orca**			+	+			49,5	7200000
**Pantholops hodgsonii**			+	+			8	33000
**Bos taurus**			+	+			20	750000
**Capra hircus**			+	+			15	45000
**Ovis aries**			+	+			22,8	93750
**Pteropus alecto**			+	+	+		15	667
**Eptesicus fuscus**	+		+	+	+		19	25
**Myotis lucifugus**	+		+	+	+		5,5	9,5
**Myotis davidii**				+	+		6	5,75
**Ceratotherium simum**				+			30	2300000
**felis catus**				+	+		14	4250
**Canis lupis**			+	+			5	51500
**Mustela putoris**							6	975,5
**Odobenus rosmarus**			+	+			35	1050000
**Ailuropoda melanoleuca**				+	+		12,5	102500
**Tursiops truncatus**			+	+			45	400000
**Leptonychotes weddellii**			+	+			30	450000
**Trichechus manatus**			+	+			55	475000
